# Patients’ perceptions of medicines information received at hospital discharge in Norway: a qualitative interview study

**DOI:** 10.1007/s11096-020-01122-0

**Published:** 2020-08-14

**Authors:** K. Svensberg, E. Trapnes, D. Nguyen, R. A. Hasan, J. K. Sund, L. Mathiesen

**Affiliations:** 1 Department of Life Sciences and Health, Faculty of Health Sciences, Oslo Metropolitan University, Oslo, Norway; 2 Department of Pharmacy, Section for Pharmaceutics and Social Pharmacy, University of Oslo, Oslo, Norway; 3 Department of Pharmaceutical Services, Oslo Hospital Pharmacy, Hospital Pharmacies Enterprise, South Eastern Norway, Oslo, Norway; 4 Department of Pharmacy, Section for Pharmacology and Pharmaceutical Biosciences, University of Oslo, Oslo, Norway; 5 Faculty of Medicine and Health Sciences, Norwegian University of Science and Technology, Trondheim, Norway; 6 Central Norway Hospital Pharmacy Trust, Trondheim, Norway

**Keywords:** Hospital discharge, Medicines information, Patient empowerment, Self-efficacy

## Abstract

*Background* Insufficient transfer of medicines information is a common challenge at discharge from hospital. Following discharge, home dwelling patients are expected to manage their medicines themselves and adequate counselling is an important prerequisite for patient empowerment and self-efficacy for medicines management. *Objective* The aim was to identify patients’ needs for medicines information after discharge from hospital, including the patients’ perception and appraisal of the information they received at discharge. *Setting* The study enrolled patients discharged from three medical wards at a secondary care hospital in Oslo, Norway. *Method* Patients were included at the hospital, at or close to the day of discharge and qualitative, semi-structured interviews were performed during the first 2 weeks after discharge. Eligible patients were receiving medicines treatment on admission and after discharge, were handling the medicines themselves, and discharged to their own home. Data were collected in 2017. Interviews were analysed with thematic analysis inspired by Systematic Text Condensation. *Main outcome measure* Patients’ perceptions of medicines information. *Results* In total, 12 patients were interviewed. They were discharged in equal numbers from the three wards, representing both sexes and a broad age range. Patients perceive medicines information as a continuum and not limited to specific encounters, like the discharge conversation. They gain information in several ways; by receiving information from health care professionals, through observations, and by seeking it themselves. Some thought they could have been better informed about adverse reactions and how to manage life while being a medicines user. Others felt they did not want or need more information. Patients employ various strategies for coping with their use of medicines, influencing their self-efficacy towards medicine management. *Conclusion* Medicines information should focus on empowering the patients throughout the hospital stay and not solely at discharge, taking into account the individual patient’s needs for information, preferences and prior knowledge.

**Electronic supplementary material:**

The online version of this article (10.1007/s11096-020-01122-0) contains supplementary material, which is available to authorized users.

## Impacts on practice

- Patients gain information in several ways while hospitalised; by receiving information and through observations. Many will also seek information themselves, when needed.
- Health care professionals should be aware that they are a source of medicines information during the hospital stay, from admission to discharge, and not restricted to when actively giving information at discharge.
- Medicines information should include practical information as well as information about how the medicines might affect the patient’s daily life. Information should be given orally and in writing.
- Rather than focusing on giving information based on their own perceptions, health care professionals should focus on revealing what the patient already knows and wants to know and use methods like teach-back, question prompt lists or goal attainment scales to improve patient empowerment.

## Introduction

Insufficient transfer of medicines information is a common challenge when patients are discharged from hospital to primary care and may negatively affect continuity of care and health outcomes [1–4]. The information is frequently delayed and discharge letters may be erroneous, e.g. by omission of medicines from the medication lists [1, 2, 5, 6]. Following discharge, most home dwelling patients are expected to manage their medicines themselves, often without the support of health care professionals [7, 8]. According to the World Health Organisation, only approximately 50% of people with chronic illness are adherent to their medicines treatment [9].

Adequate counselling is an important prerequisite for patient empowerment and self-efficacy for medicines management [10]. Self-efficacy, defined as the patient’s conviction that one can successfully execute the behaviour required, is strongly correlated to empowerment, and both concepts are connected to adherence [10–12]. Previous studies have shown that patients’ information needs are individual [13, 14]. Whereas some patients want information, especially concerning adverse reactions and drug interactions, some prefer not to be informed [11, 13]. Methods to tailor medicines information to the individual patient could include teach-back methods and goal attainment scales [15, 16].

While conducting a previous study on the effect of a pharmacist-led intervention in hospitalised patients, we experienced the discharge process as complex, as has previously been described by others [17]. We designed the current study to gain better insight into patient’s experience of medicine information at discharge from hospital, aiming at defining an optimal process with respect to medicines information. Although the importance of adequate medicines information has been thoroughly documented, there are relatively few publications based on interviews with patients after discharge. Better insight into patients’ perception and actual need of medicines information in order to increase self-efficacy could contribute to improved and better tailored interventions.

## Aim of the study

The aim was to identify patients’ needs for medicines information after discharge from hospital, including the patients’ perception and appraisal of the information they received at discharge.

## Ethics approval

The Regional Ethics Committee found no ethical approval necessary. The study was approved by the Privacy Ombudsman and the Hospital Investigational Review Board June 13, 2016, Reference Number 2016/9269.

## Method

### Study design

This was a qualitative study individually interviewing patients discharged from three medical wards at a secondary care hospital in Oslo, Norway [18]. The interviews were performed by two master students in pharmacy, authors RAH and DN.

### The Norwegian context

Hospitalised patients receive all their medicines from the hospital. However, medicines are not dispensed at discharge, and patients are rarely receiving counseling by a pharmacist at the time of discharge. Thus, home dwelling patients, responsible for handling their medicines, will have to visit a pharmacy to collect their prescriptions after discharge. For medicines initiated at the hospital, the hospital physician provides prescriptions. The discharge summary, including an updated medication list, is handed to the patients, as well as forwarded to the GP, and if relevant, to the home care nurse. The GP has the overall responsibility for patients when they are not hospitalised.

According to Norwegian legislations, the patients are entitled to receive the information necessary to understand their situation, including possible risks and adverse reactions. The information should be adapted to the individual, and the Health Care Professionals (HCPs) should ascertain that the patient has understood it [19, 20]. This applies to the General Practitioner (GP), the HCPs at the hospital and at pharmacies. In Oslo, the patients’ last discharge information is available to the patient through a web page, called “My Medical Records” (Norwegian: Min Journal, https://www.minjournal.no).

### The interview guide

A semi-structured interview guide was developed based on the aim of the master theses and clinical experience (supplementary material). Input from a representative from the User’s Board at the Hospital Pharmacy Enterprise, South Eastern Norway ensured that the topics covered were relevant from a patient’s perspective (content validation). The guide was evaluated after first interviews, and as no major changes were necessary, these interviews were included in the analysis. The aim of the master theses was to describe patients’ perception of the medicines information received at discharge from hospital in order to identify their need for medicines information, and furthermore, to study the patients’ actual use of medicines after discharge. Prior to the analysis presented in the current paper it was decided to exclude the last part of the aim, i.e. the patients’ actual use of medicines, as these results were more quantitative in nature and less informative for a thematic analysis.

### Data collection and analysis

Patients were recruited at discharge from medical wards, either internal medicine, nephrology or cardiology. The wards admit multimorbid patients of all age groups, patients with kidney diseases and with resistant blood pressure, and patients suffering from acute or chronic heart diseases, respectively. The study was conducted by two consecutive master students, and the inclusion period was March to November 2017. Purposive sampling was used and maximal variation strived for with respect to hospital wards and the patients’ sex and age [18]. Patients were eligible for inclusion if they were regularly using medicines both at admission and discharge, were handling the medicines themselves, were residents in Oslo and discharged to their own home, had adequate knowledge of Norwegian, and were able and willing to provide a written informed consent. Terminally ill patients were not eligible.

Nurses dedicated to assist in the study informed about planned discharges, and eligible patients were recruited, and informed consent retrieved by authors RAH and DN. The participants were contacted by telephone to schedule the interview, preferably during the first 2 weeks after discharge. The interviews were conducted by authors RAH and DN at visits to the participants’ homes or, if the participant preferred, at the hospital during a control visit. The interviews were audiotaped and transcribed verbatim by DN. Participants received a gift card with a value of 200 NOK.

The interviews were firstly analysed by author DN and presented in her master thesis [21]. We present a reanalysis of the results to gain new perspectives of the medicines information related parts of the transcripts [22]. This inductive thematic analysis, inspired by Systematic Text Condensation was conducted by authors LM and KS (pharmacists in academia), and ET and JKS (clinical pharmacists) [23]. The transcripts were independently read, and themes derived inductively, first individually, and thereafter at three consensus sessions. During the final interpretation process, the concepts of coping strategies were introduced (Table 1). People use coping strategies to manage the demands of stressful events, like hospitalisations [24, 25]. Such strategies include both cognitive and emotional processes [26] and have been presented as several dimensions (Table 1) [24]. The results were therefore analysed according to this framework, with coping emerging as a main theme.

**Table 1:** Coping strategies

Strategies^a^	Description	Examples
Planful problem-solving	Deliberate problem-focused efforts to alter the situation, coupled with an analytic approach to solving the problem	“I made a plan of action and followed it”, “Just concentrated on what I had to do next—the next step”
Accepting responsibility	Acknowledge one’s own role in the problem	“Realised I brought the problem on myself”
Confrontive strategies	Aggressive efforts to alter the situation, as well a degree of hostility and risk-taking	“Tried to get the person responsible to change his or her mind”, “Stood on my ground and fought for what I wanted”
Seeking social support	Efforts to seek informational, tangible or emotional support	“Talked to someone to find out more about the situation”, “I asked a relative or friend I respected for advice”
Distancing	Efforts to detach oneself and to create a positive outlook	“Looked for the silver lining, so to speak; tried to look on the bright side of things”

^a^All the information given in the table, including the examples, is taken from Folkman et al. [24]. Only the strategies relevant for this paper have been included

## Results

In total, 26 patients were invited, whereof 19 gave informed consent. After discharge, the authors were able to get in contact with 12 patients to perform the interviews. The participants were discharged in equal numbers from each of the hospital wards. Males and females were equally represented, as well as a broad age range (Table 2). The interviews lasted between 16 and 66 min.

**Table 2:** Demographics of the study population

Hospital ward	Internal medicine	Nephrology	Cardiology	Total
Female/male (n)	2/2	2/2	2/2	6/6
Age, median (range)	67 (23–73)	67 (39–80)	60 (47–91)	65 (23–91)
Number of medicines at admission, median (range)	6 (1–15)	12.5 (7–13)	4 (0–11)	9 (0–15)
Number of new medicines at discharge, median (range)	1.5 (0–2)	1.5 (0–3)	1 (1–4)	1 (0–4)

To enrich the analysis, we present narratives from two participants, presented with pseudonyms (Table 3). The first narrative describes a patient who had gone through a major cardiovascular event and had limited previous experience with medicines. He had questions to his treatment and described coping strategies as planful problem solving and seeking social support. The second narrative, had been using medicines for several years and did not want to be too preoccupied with her medicines, reflecting a distancing strategy, but at the same time she also gave examples of planful problem solving.

**Table 3:** Two patient narratives representing unique views and perceptions on medicines information

Karl	Karl was hospitalised after an acute myocardial infarction, and had a percutaneous coronary intervention. He had previously been well and considered the event a big change to his life. He felt that he had been well informed throughout the hospitalisation about the medicines he received
Male, 61 years old	“During the transport to the hospital the paramedics continuously told me what they were doing and what effect it should have… Similarly, on arrival at the emergency department they said “you are now getting an Aspirin dissolved in some water”… So I have been well informed about what they put into my body”
Limited experience with regular use of medicines	He was familiar with some of his medicines in advance, as his mother had been using the same for some years. With respect to written information, a nurse had shown him a ring binder with information and told her it was a good idea to take pictures of it with his smart phone However, he thought that he had not received enough information about possible adverse reactions, and how and when to take the medicines. He was not certain about the dosing of the medicine until after he had collected them at the pharmacy “…when I was there (at the hospital) I didn’t ask any of the nurses about adverse reactions, because when I lay there, I wasn’t dizzy or anything” He also felt that he was not well informed about what treatment effect he could expect, or how the treatment might influence his daily life “… I have been thinking…I go for a walk every day, and have been told to do so, but I am uncertain about how much activity I can endure. Because when I walk, and especially walking up hills, I get a bit sweaty and I know someone also using metoprolol who had been told that if he started to sweat, he should stop, but I was not told anything about that. I will ask my GP about this when I see him”
Anne Female, 89 years old	Anne is married and living with her husband. She has been using medicines for several years. She had received information about her new medicines at the hospital, but considered herself experienced and that she did not really need any information. She could not really recollect having received detailed information from the hospital, but thought this was due to the HCPs evaluating her fully able to cope. She did not miss any information from the hospital, and said she found it sufficient to read the PIL. Anne even considered it was not good to be too occupied with your medicines
Had used medicines for many years	“…I can’t remember just now…what it’s meant for. Actually, I don’t walk around thinking about it, I’d rather think about pleasant things. You might be concerned about this if you are using one or two drugs. Then you might remember exactly why you are taking them. But using nine drugs…I don’t think it’ll do you any good to remember why” What was important to Anne was to be able to control her stock of medicines at home. This was important because at discharge from hospital you get this list of medicines, and you go to the pharmacy to get you medicines, and when you come home you realise some of them are the same as those you already have. Accordingly, she made her own system on her PC. “So I made this system, and when I empty one box, I can check my list to see what I have in stock”

*HCPs* health care professionals, *PIL* patient information leaflet

In the thematic analysis, the following main themes emerged:

1. The patient’s conceptualisation of medicines information—a constant flow of information.
2. Ways of coping with being a medicines user.

The main themes were further divided into subthemes.

### The patient’s conceptualisation of medicine information: a constant flow of information

We aimed to describe patients’ perception and appraisal of the medicines information they received at discharge from hospital, as well as to identify their information needs after returning home. However, the patients did not share our understanding of medicines information at discharge. Rather than seeing this as a defined concept, they included all medicines information received from the ambulance and throughout the hospital stay. Furthermore, their conceptualisation of medicines information comprised other sources of information and even a general trust in the health care system, including the development and approval of medicines.

#### Medicines information at the hospital

The participants described various ways of gaining medicines information at the hospital, such as oral or written information given by HCPs, or by observing HCPs’ routines. The participants emphasised the importance of written information, which can be reread after arriving home.

I did get an updated list of medicines. She handed me one such print out, and that’s fine… I think that’s important, because you might not get everything they say when you are lying there, are a bit under the weather, and might be hurting a bit here and there. So…my point is it’s important that you get something in writing—that’s worth its value in gold… (Man, 70)

The written information comprised the medicines list in the discharge summary, and information leaflets or binders. The binders were described as copious, comprising information about the illness as well as the medicines. The participants reflected that during the hospital stay it was quite busy, and they received a lot of information. At the same time, they were not well, making it difficult to absorb oral information. Several participants shared a positive experience of HCPs having gone thoroughly through the written information, checking the patient’s recollection of the information. Others expressed the lack of such a perusal, which they thought could have opened for the possibility for follow-up questions.

#### Medicines information needs

The participants reflected on various aspects of the medicines information, related to both content and form. Several of the participants considered they had not been sufficiently informed about adverse reactions. Furthermore, they found that medicines information should include information about how to manage life while being a medicines user, and what treatment effect to expect. In their opinion, the HCP’s information focused on the cause of the hospitalisation, which drugs to take and why. However, they wished to be better informed about how medicines could influence their daily living, e.g. their ability to exercise. Some participants wished to receive more information about drug–drug interactions as well as interactions with food.

Several of the participants knew about “My Medical Records” online, and one had chosen to receive written information only in this way. Others considered they did not possess sufficient IT-skills and preferred to have a paper to hold and read. Some of the participants reflected that the language in the discharge information sometimes would be rather unavailable to lay people, e.g. using Latin words like “vesp” (at night).

#### The importance of trust and respect

The participants underlined the importance of being met in a good way by HCPs. They wanted to be treated respectfully and listened to, and contemplated on how meta-communication from the HCPs had affected the conversation, e.g. placing oneself in the same height as the patient.

Most doctors would sit at your bedside, them being up there and me being down there. What was good with this one was that he placed himself down with me so that we were at the same height. I liked that. Very much. (Man, 64).

Trust was important to the participants to be able to manage their medicines use. Trust towards the General Practitioner (GP) and the hospital physician was emphasised as necessary, as well as towards the medical profession as such. Several participants thought physicians were very competent, able to decide on correct treatment, and able to check that there were no drug interactions. They also trusted that the GP received the discharge information from the hospital and that she would take responsibility for an adequate follow-up.

Some participants said that they had great confidence in the system for development and approval of medicines, especially the Medicines Agency. They trusted that medicines were appropriately tested, and the fact that many others are using the same medicines added to the feeling of security.

### Ways of coping with being a medicines user

#### Planful problem-solving

The participants were generally well motivated to take their medicines and reported various ways of seeking the information they needed to cope with being a medicines user. They had actively sought information during hospitalisation, or even prior to admission, by asking HCPs about their treatment. Some of them said they observed the handling of medicines at the ward and reflected on how this could be translated into their own handling of medicines after returning home. They found it confusing when the hospital’s routines deviated from how they perceived it should have been.

When I was hospitalised, I received intravenous antibiotics every fourth hour. […]… They have routines giving medicine at 6 o’clock, and then every fourth hour. The day I was discharged, I got intravenous drug at six, but I didn’t get the tablet until two. So the four hours weren’t that important after all. (Woman, 39).

Participants who felt they had not received adequate information at discharge had logged into “My Medical Record” to check. Participants also told that they sought information on the internet to add to the information received at the hospital, or they would have done so if they had needed to; some told that they used to “google”, whereas others used the lay version of the national drug formulary. To them obtaining such information meant less need to contact HCPs. In addition, many participants considered the GP to be an important source to medicines information; they had gathered up questions to bring to their next appointment. Several participants had sought information at the pharmacy, where they received information about practical issues such as dosing and administration, as well as drug interactions.

#### Accepting responsibility

Many participants mentioned the Patient Information Leaflet (PIL) as a very important source of information. In the PIL they found vital information like the indication and side effects. They thought medicines users had an individual responsibility to read it.

Here, you see? It (i.e. the PIL) gives information about side effects, what it (i.e. the drug) is meant for. It’s like, if you don’t read it (i.e. the PIL), then you have nothing to complain about (Man, 80).

#### Confrontive strategies

Several participants experienced receiving inaccurate information, leading to misunderstandings and frustrations. One participant with complex morbidities perceived the HCPs as insecure, frequently conferring specialists at another hospital. The participants reflected that the lack of communication between physicians involved in their treatment at the hospital, lead to an apparent lack of a treatment plan and errors, which they had sorted out by confronting the HCPs.

Other participants had experienced that HCPs replied to questions in such a vague manner that the answer made no sense to them and they had to put the HCPs in their place.

The doctor told me nothing and when I asked her, she replied “as expected”…In the end I told her “I have no idea what you are expecting, and I would like an answer to my question”. […] after a while, she might have been given a hint, she tried her best to answer me (Man, 80).

Some participants were dissatisfied with the care and follow-up from their GP and contemplated applying for a transfer to a different GP.

#### Seeking social support

The participants reported they would sometimes seek social support from family and friends for medicines information. In addition, they would use their own experience with family members’ use of medicines, e.g. parents who used the same medicine, exemplified with the narrative Karl (Table 2). They considered this could provide knowledge on how the medicines were meant to be used, and what could possibly be adverse reactions. Some had family members or friends who were HCPs, whom they could contact if needed, reducing the need to contact the hospital. Another way of seeking social support was becoming a member of a patient organisation.

#### Distancing

Several of the participants said they had been using numerous medicines for several years, had adequate knowledge about the medicines, and did not need any more information. They would rather seek more information themselves, if ever needed. At the same time, some of them considered that it was not a good thing to know too much about the medicines, i.e. to be over-concerned about it, exemplified with the narrative Anne (Table 2). One should rather trust the physicians.

…no, I would rather not be thinking about that, I’d rather think about pleasant things…[…]… using nine medicines, I don’t think you ought to be thinking about it (your medicines)…[…] When I get new medicines I receive information and then forget about it, I don’t think about it anymore (Man, 91).

## Discussion

This study aimed to explore the patients’ information needs after discharge from hospital, and their perception and appraisal of the information received at discharge. Two main findings were revealed. Firstly, patients perceived medicines information during hospitalisation as a continuum, not limited to isolated events of HCPs actively giving information at discharge. Secondly, the patients reported the use of several coping strategies for dealing with being a medicines user.

The first main finding highlights medicines information as a continuum, not limited to information sessions at discharge. It was even perceived to include contextual factors, like general trust in the health care sector. The participants expressed a wish for both oral and written medicines information, which is in line with reviews concluding that the combination is more effective in improving knowledge and satisfaction than verbal information only [27, 28]. Acknowledging that medicines communication happens throughout the hospital stay, as perceived by the participants, and is not limited to the HCPs’ initiated sessions at discharge would be in line with the concepts of person centred care [29]. Furthermore, the participants also appreciated HCPs going through the information with them; in line with using teach back methods [15]. They also suggested that the information could have been less retrospective and rather focus on supporting their self-efficacy after discharge.

Strategies to empower patients and strengthen person centred care in relation to medicines information has previously been called for [13, 30]. This could be achieved by strengthening medicines communication throughout a patient’s stay, and tailoring it to the individual patient’s needs with respect to form, content and timing. This would also relate to the use of electronic versions of the written discharge information, as some participants in our study did not consider themselves to possess sufficient IT-skills to access such information.

The second main finding was the importance of coping with being a user of medicines. Coping strategies for “planful problem solving”, were commonly reported in our study, comprising active information seeking from the GP or at the pharmacy, as well as observing the medicines handling at the ward. Interestingly, the latter strategy complies with social learning theories having a long history in the literature on health care educations, but seemingly not commonly discussed in the context of medicines information [31, 32].

“Confrontive strategies” were also reported by participants in our study. This included criticising the care received at the hospital, or demanding a more precise information. Lack of clarity in the information received could exacerbate the health crisis which needs to be coped with [33]. Hence, insufficient information, lack of clarity, or misunderstandings, could explain the perceived need for a confronting behaviour to achieve improvements.

“Seeking social support” was an important coping strategy for several of the participants in our study. This coping strategy includes seeking informational, as well as emotional, support [24]. In our study, this comprised support from close family or friends who were either experienced users of medicines or HCPs, which is in line with previous studies [34, 35].

However, some participants expressed the opinion that they did not want or need information, saying that focusing too much on your medicines was not “good for you”, i.e. expressing “a distancing coping strategy”. This contradiction in the need for information is in accordance with previous studies, in particular related to information about adverse reactions [11, 13, 36]. Surprisingly, participants expressing such views in our study had used medicines for many years, and were assumingly already inclined to have a high degree of self-efficacy. They were also of older age, which has previously been found to be associated with a lower wish for information [37].

In summary, the strategies reported are considered to be problem focused forms of coping, found to dominate when people appraise the situation as changeable [24, 33]. These strategies could be considered important to promote self-efficacy in managing medicines and patient empowerment [10, 38].

Our results emphasise that HCPs need to be aware that medicines information is mediated continuously in various ways, i.e. passively (behaviour) and actively (any information given by any HCPs). Thus, rather than solely focusing on the discharge conversation as the main encounter for giving medicines information, HCPs should focus on empowering the patient throughout the hospital stay. HCPs should also raise their level of awareness of medicine users’ coping strategies as this could strengthen their ability to work more patient centred, by understanding individual needs and adapt their behaviour accordingly. This is supported by the fact that participants in the current study employed coping strategies compatible with wishing to take responsibility for their medicines use, which is in line with previous studies [30]. It is therefore important to appreciate and strengthen the patient’s role in medicines information and for HCPs to actively involve the patients in the treatment, to enhance the empowerment and self-efficacy towards medicines use after discharge [13, 30]. It also underlines that the information should focus more on ensuring patient motivation or skills, e.g. using teach back methods [15, 16, 39]. Furthermore, the question “what matters to you?”, which is considered essential to patient centred care [40], could also be implemented for medicines information.

We propose that a medicines information diary could be developed for patients who are responsible for handling their own medicines. It would resemble diaries for symptom monitoring and adherence, which have also been shown to increase patient participation [41]. This diary could be used by the patient to facilitate the communication with HCPs, as well as to visualise the information continuum for various HCPs involved in the care, and to collect the written information received. The diary should be combined with teach-back methods to identify the patients’ understanding of their medicines treatment, or solve any misunderstandings. It could also be combined with the use of Question Prompt Lists or goal attainment scales [16, 42].

Future research should aim to provide further understanding of the medicines communication between HCPs and the patients throughout the hospital stay including discharge, e.g. by observing such encounters. It should also study interventions like the suggested diary, in which the information is tailored to the individual patient’s needs and how these might influence patients’ self-efficacy in managing medicines and adherence.

Limitations commonly associated with semi-structured interviews apply for our study [18]. It is supposed that participants will feel less disempowered if the interviews are conducted outside the hospital setting [43]. However, we experienced that patients expressed reluctance to invite the study pharmacist to their home, which might have been a reason for refusal to participate. Thus, we might have recruited the most empowered and confident patients with high self-efficacy towards medicine management. On the other hand, we succeeded in recruiting a broad sample. We believe this resulted in a higher information power and that we further have contributed to new aspects of our study aim [44], especially regarding the number of coping strategies identified. The inclusion criteria did not focus explicitly on patients receiving new medicines, which might be considered a limitation. One participant used no medicines at admission. Nevertheless, as the interview contributed interesting data it was included in the analysis. The interviewers were inexperienced in conducting interviews, which also might have been a limitation. The interviewers were not involved in the care of the patients at the hospital, however, participants may still have given socially desirable answers, associating the interviewers with their hospital stay.

## Conclusion

Medicines information should focus on empowering the patients throughout the hospital stay and not solely at discharge, taking into account the individual patient’s needs for information, preferences and prior knowledge.

## Electronic supplementary material

Below is the link to the electronic supplementary material.

Supplementary material 1 (DOCX 20 kb)
