# Supplementary material for: Patients’ perceptions of medicines information received at hospital discharge in Norway: a qualitative interview study
Source: Int J Clin Pharm. 2020 Aug 14;43(1):144–53. doi: 10.1007/s11096-020-01122-0 (PMC7878245; doi:10.1007/s11096-020-01122-0)
Supplement: Supplementary file 1 — Supplementary material 1 (DOCX 20 kb) [file 11096_2020_1122_MOESM1_ESM.docx]

# Supplementary material

“Patients' perception of medicine information received during hospitalisation - a qualitative study”

International Journal of Clinical Pharmacy

Authors: Svensberg K^1, 2^, Trapnes E^3,4^ Nguyen D^4^, †, Hasan RA^3, 5^, Sund JK^5, 6^ and Mathiesen L^4^

1. Department of Life Sciences and Health, Faculty of Health Sciences, Oslo Metropolitan University, Oslo, Norway

2. Department of Pharmacy, Section for Pharmaceutics and Social Pharmacy, University of Oslo, Oslo, Norway

3. Department of Pharmaceutical Services, Oslo Hospital Pharmacy, Hospital Pharmacies Enterprise, South Eastern Norway, Oslo, Norway

4. Department of Pharmacy, Section for Pharmacology and Pharmaceutical Biosciences, University of Oslo, Oslo, Norway

5. Faculty of Medicine and Health Sciences, Norwegian University of Science and Technology, Trondheim, Norway

6.Central Norway Hospital Pharmacy Trust, Trondheim, Norway

† Deceased

Corresponding author: Liv Mathiesen, livmathi@farmasi.uio.no

## The interview guide

### Introduction to the interview

- Repeat the information about the study (given when collecting informed consent).
- Emphasis that what is said during the interview will by anonymised in the published text and that no statement can be linked backwards to the patient.
- This will be a conversation about your experiences about receiving medicines information. Although I am a pharmacy student, in this respect I am the interviewer and cannot provide any information or advice about your treatment. If you have any questions, I will advise you to talk to your general practitioner.
- The interview will normally take approximately 45 minutes
- The conversation will be audiotaped

### Start of interview/General overview

1. How has taking your medicines been after discharge from hospital?

Examples of follow-up questions:

- Can you give any examples of what has been good / bad with using your medicines?
- Can you give any details about why this was good/bad?

### Medicines information

1. Did you experience changes to your drug treatment at the hospital?

Examples of follow-up questions:

- Which changes were done? (New medicine, dosing etc)

1. How was the information provided? Orally or written?

Examples of follow-up questions:

- Can you tell more about what kind or oral information you got?
- Can you tell more about the written information and what it contained?
- When did you read the written information (immediately after you received it, or did you wait?)

1. Did you receive information about the medicines you were already using prior to admission? Can you tell something about what kind of information this was?
2. Did you receive information about the new medicines you started up with at the hospital? Can you tell something about what kind of information this was?
3. Did you receive any information about how to administer your medicines? Can you explain to me how your tablets are supposed to be taken (e.g. with/without food?/How to use the equipment (if relevant))?
4. How would you assess the medicines information you received when you were discharged from hospital?

Examples of follow-up questions:

- Please give examples of what you think was good / bad about the information
- Who gave this information?
- Please give examples of what could have been done differently?
- Please give examples of how you would have liked the information to be
- Was there something you think was lacking in the information?

### Follow-up after discharge

1. Did you receive any assistance to assure that you could continue with your treatment as scheduled after discharge?

Examples of follow-up questions:

- Can you tell more about what kind of help you received?
- Were you able to immediately collect your prescription at the pharmacy?
- Did you receive any assistance to collect your prescription?

1. Have you been able to use your medicines daily after being discharged?

Follow-up question:

- Could you explain why it has not been possible?

1. Have you been in contact with your general practitioner or the hospital after the discharge? Do you know what kind of follow-up might have been planned with respect to your medicines?
2. How do you think the follow-up with respect to the treatment with medicines should be when patients are discharged from hospital?

### The use of medicines

1. Can you please explain to me how you use your medicines? You might show me your medicines if you are not able to remember the names of your medicines.

Follow-up question:

- Can you tell me what this drug is meant for (why you are using this drug)

1. Which medicines list is the one you are adhering to for your treatment? (The GPs, the one from the discharge letter)

Follow-up question (if not the list from the discharge letter):

- Can you tell why you are using this list?

1. What do you do to remember to take your medicines?

Examples of follow-up questions:

- Has it at any time happened that you have forgotten to take your medicines?
- When did it last happen?
- What do you do when you realise you have forgotten?
- Have you received any advice from the hospital about how to remember to take your medicines?
- Do you receive any assistance in handling your medicines? From whom? And what kind of help do you receive?

1. Have you wondered about anything regarding the use of your medicines after you were discharged from the hospital?

Follow-up:

Related to

- How to take the medicines?
- Adverse events?
- What treatment effect to expect?
- Use of any equipment?

1. What do you do if you have any questions related to your treatment with medicines?

Examples of follow-up questions:

- Who would you contact if you had any question?
- Would you say you feel safe regarding the use of your medicines?

1. To sum up, can you repeat what you were pleased with and not so pleased with respect to the medicines information you received at discharge from hospital?
2. Is there something you would like to add, or something you have remembered during our conversation?
